# Revisiting the concept of entomotoxicology

**DOI:** 10.1016/j.fsisyn.2020.09.003

**Published:** 2020-09-28

**Authors:** Jiri Hodecek

**Affiliations:** University Center of Legal Medicine Lausanne – Geneva, Swiss Human Institute of Forensic Taphonomy, Chemin de La Vulliette 4, CH-1000, Lausanne 25, Switzerland

**Keywords:** Entomotoxicology, Forensic, Environmental, Entomology, Toxicology, Xenobiotics

## Abstract

Until now, the term *entomotoxicology* has only been used in medico-legal sciences. However, *entomotoxicology* as a whole has a much wider scope and *forensic entomotoxicology* is just one of its branches. Based on the literature a wider definition of the term is presented. Today, we can distinguish two major branches of *entomotoxicology*: 1) *Forensic entomotoxicology*, which uses the insects as evidence of the presence of xenobiotics in decomposing tissue during an investigation and 2) *Environmental entomotoxicology*, which uses the insects as bioindicators of environmental pollution in non-criminal circumstances. While *forensic entomotoxicology* is a relatively new discipline, research in *environmental entomotoxicology* began as far back as the 1920s. A review of the work in *entomotoxicology* from the last 6 years is presented, covering several interesting new trends. This article aims to redefine entomotoxicology, which should increase awareness to bring more collaborations and multidisciplinary between related scientific fields.

## 1 Introduction

Bioaccumulation of toxins in insect bodies is well known [[1], [2], [3]]. The presence of certain xenobiotics (e.g. drugs, insecticides, repellents, and heavy metals) in a given environment, such as landscape, river, or carrion, can play a crucial role in the analysis of such habitats. In some cases, it is suitable or even inevitable to use insects as evidence of such pollution. Insects can help us detect xenobiotics but can be also used as models to study the effect of specific xenobiotics on target insect species. As xenobiotics can vary significantly, so do the aims of the particular studies, which are focused on them. However, all such studies belong to the broad scope of *entomotoxicology*.

The word *entomotoxicology* is a compound of words from ancient Greek: “entomon” meaning insect, “toxikos” meaning poisonous and “logos” meaning subject matter. Therefore, etymologically, it is a field studying xenobiotics affecting insects. The term was firstly used by Pounder in 1991 [4]. Pounder [4] used it with caution adding the adjective “*forensic*”, which by itself should indicate a broader definition of the original term. However, until now, no one particularly identified the missing part of the scope of *entomotoxicology*, and this term was used solely for forensic sciences. There are many studies within the scope of *entomotoxicology*, which were never considered “entomotoxicological” before, even though they focused on the xenobiotic accumulation in insect bodies and their biological effects. Due to the inexplicit terminology, the opportunities for collaboration between different fields are being diminished and overlooked, which leads to further isolation of otherwise scientifically related fields. The specification of the terminology could, therefore, bring: 1) standardization of methodology in *forensic entomotoxicology* and 2) increased knowledge about the effects of pharmaceuticals and illegal drugs on insects and their environment in *environmental entomotoxicology*.

In this article, a new definition and use of the term *entomotoxicology* are proposed based on the available literature. To better characterize the scope of the contemporary field, recent research, particularly from 2012 onwards is the focus of this review. The review of entomotoxicological research focused on the effects of insecticides and repellents on insects, the bioaccumulation of heavy metals in insect bodies, and the traditional studies in the forensic sciences are presented.

### 1.1 The new fields of entomotoxicology

Campbell [5] proposed the term “Insect toxicology” as a new term for all investigations of the effects of insecticides on insects already in 1926. Insect toxicology should be a synonym of the term *entomotoxicology*, however, it had not been used that way before. Pounder [4] defined *forensic entomotoxicology* as the detection of drugs present in a decomposing corpse via analysis of fly larvae (“maggots”) feeding of it. The term *entomotoxicology* became popular and nowadays almost all articles focusing on research of toxins in insect bodies for medico-legal purposes use it. Nonetheless, the definition itself came through a subsequent development: In 1994, Goff and Lord [6] defined *entomotoxicology* as the use of insect specimens for toxicological analyses in the absence of tissues and fluids normally taken for purposes of death investigations. However, they did not add the adjective *forensic* and therefore suggested that *entomotoxicology* concerns only medico-legal approach. Many other authors repeated the same approach after them [[7], [8], [9], [10], [11], [12], [13], [14], [15], [16], [17]], while other authors specified their field as *forensic entomotoxicology* [[18], [19], [20], [21], [22], [23]]. The most suitable terminology was used by da Silva et al. [19], who defined *forensic entomotoxicology* as the use of insect specimens as an indirect source of toxicological evidence in the absence of direct matrices, such as blood, urine, soil or water, in determining the presence of a xenobiotic in the environment (which may be a dead body, a river or even an entire landscape). Da Silva et al. [19] divided *forensic entomotoxicology* into 1) *environmental forensic entomotoxicology*, which emphasizes the use of insects as bioindicators of environmental toxicants and 2) *medicolegal forensic entomology*, which focuses on using insects as surrogate or proxy samples when bodies are too decomposed to provide toxicological samples. In da Silva’s article, there is a tendency to define the field more precisely, however like most other authors before them, they considered *entomotoxicology* only within its medico-legal borders. Campobasso et al. [18] already used the terminology suggested by da Silva [19] and underlined that insects can be used also as direct evidence of heavy metal pollution near industrial areas or antidepressant contamination of river ecosystems during such investigations.

However, the field *entomotoxicology* logically contains not only research for investigational purposes, but also all other types of toxicological examinations where insects are studied (e.g. effects of insecticides and repellents on insects, or heavy metal accumulation in insect bodies). The scope of *entomotoxicology* is thus much wider than previously acknowledged. Some of its main aims include addressing: 1) the possibility of particular xenobiotic detection; 2) the quantitative relationship between the amount of the xenobiotic obtained from an insect body with the amount detected in the insect’s diet and/or the environment; 3) the influence of the xenobiotic on the growth, morphology and overall fitness of studied insect and 4) the testing of different methods and approaches for xenobiotic detection. In current terminology, research of a non-investigational character is usually addressed as “ecotoxicological” [18,24,25], while some authors still use Campbell’s “insect toxicology” [26]. The term “ecotoxicology” is valid and it already includes *entomotoxicology*. However, *entomotoxicology* should be used as a more precise way of describing ecotoxicological research, which targets insects. A new definition of the term *entomotoxicology* is therefore proposed: *Entomotoxicology* analyses the effects of xenobiotics on insects and uses xenobiotics present in insect bodies as evidence of environmental pollution. The environment can be pictured as carrion, soil, river, landscape, or even the body of a patient (according to the clinical applications).

**Fig. 1:**
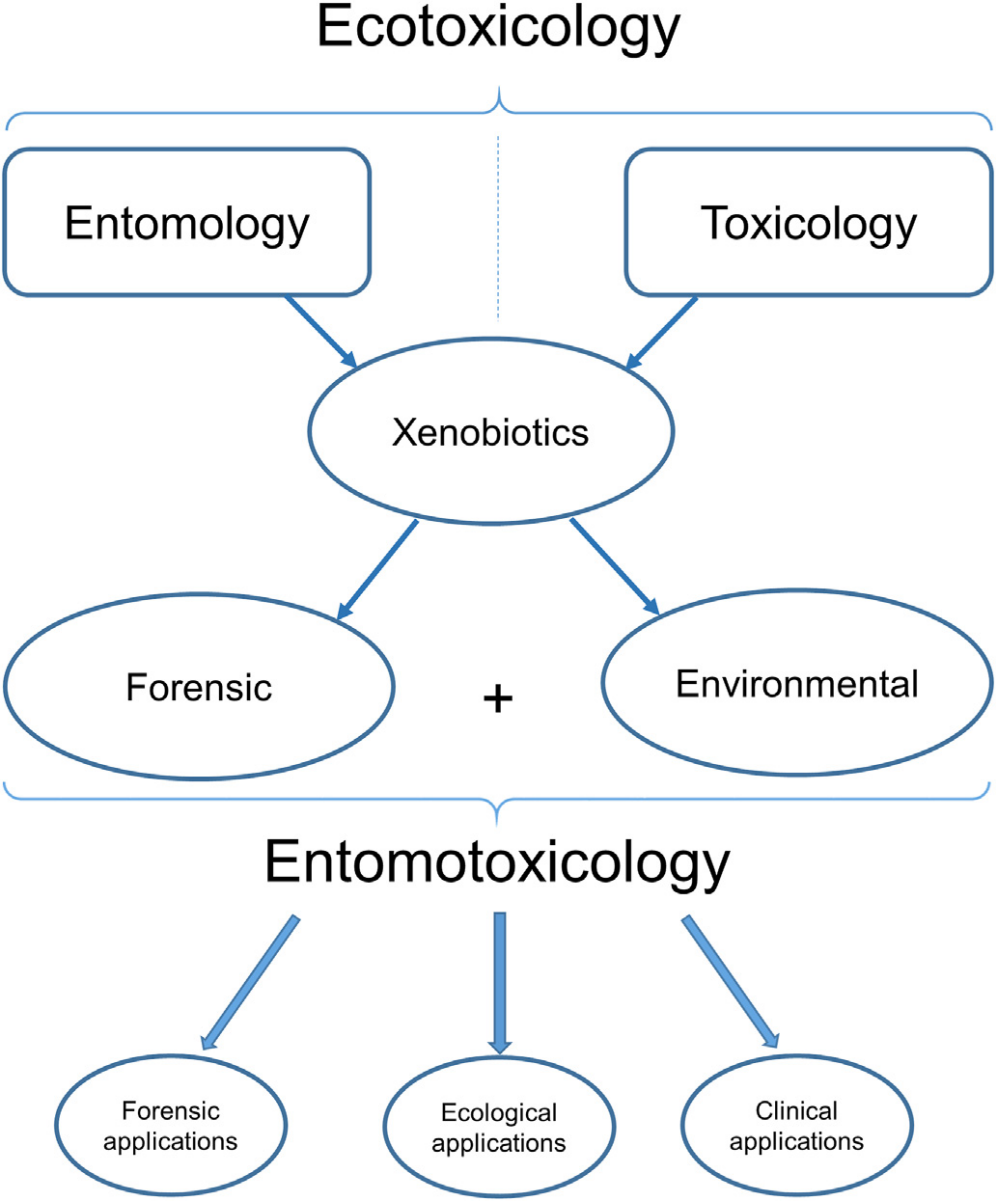
Reorganisation of entomotoxicology concept: Entomotoxicology is a subfield of ecotoxicology and can be divided into forensic and environmental entomotoxicology based on the outcomes.

Based on the study approach, the field can be divided into *forensic entomotoxicology* and *environmental entomotoxicology* (Fig. 1). *Forensic entomotoxicology* includes all studies with medico-legal applications. When the outcome of the study is to bring new evidence for ongoing investigation or to improve its methodology, it is a part of the medico-legal sciences (this does not necessarily contain only investigations of human remains - cf. da Silva’s medicolegal/environmental *forensic entomotoxicology* [19]). While *forensic entomotoxicology* is always a part of medico-legal entomological studies, *environmental entomotoxicology* has a wider area of applications. The outcomes of *environmental entomotoxicology* are mostly ecological, but some research may include medical applications (e.g. maggot/larva therapy). In these cases, the “environment” may be represented by the habitat the insect is living in, i.e. a living human body for clinical applications and a dead human body for medico-legal applications.

### 1.2 Forensic entomotoxicology

In forensic entomology, insects have been used for xenobiotic detection for about 40 years [27,28]. *Forensic entomotoxicology* is, therefore, a relatively new branch of *entomotoxicology*. *Forensic entomotoxicology* focuses mainly on determining the effects of different xenobiotics on various insect species of medico-legal importance. In cases where certain xenobiotics are present, there is an increased risk of the post-mortem interval (PMI) estimation bias, because some xenobiotics can alter the developmental time of necrophagous insects [10]. Moreover, different species of necrophagous insects can react differently to the same type of xenobiotic [20]. Another important aim of *forensic entomotoxicology* is the determination of the cause of death [13,[18], [19], [20]].

From 1977-2017 only 63 scientific articles in *forensic entomotoxicology* have been written [19]. Although the importance of *forensic entomotoxicology* for PMI estimation and the cause of death determination can be essential, the field is still considerably understudied. A number of limitations and inconsistencies between the studies have been revealed by da Silva et al. [19]: 1) A lack of methodological standardization is necessary to enable comparisons between studies; 2) Most studies have insufficient numbers of replicates, which increases the risk of statistical inference errors; 3) Successful entomotoxicological study can be expensive which means validation against broader toxicological standards is often not possible; 4) Most studies were conducted either only by an entomologist or a toxicologist. However, both entomological and toxicological approaches are equally important and have to be carefully validated so that vital information is not overlooked. Campobasso et al. [18] also concluded that one of the main weaknesses of the field is the correlation in between the xenobiotic levels in larvae with the amount detected in the cadaver. The interpretation of results is also handicapped by our poor knowledge of the metabolism of xenobiotics in insect bodies, and of the unknown impacts of environmental factors on xenobiotic detection, different drug concentrations in different tissues and uneven sampling efforts [18,20]. Furthermore, laboratory animals can produce different metabolites than humans, which may lead to some incorrect assumptions [12].

### 1.3 Environmental entomotoxicology

*Environmental entomotoxicology* is more extensively studied than its forensic counterpart. One of the most studied branches of *environmental entomotoxicology* is the effects of insecticides on insects. A lot of work has been done since Campbell [5], but it will always be a little bit of an “arms race” between a newly developed insecticide and the research of its impact on the organisms and their environment. Neonicotinoids and fipronil are among the most widespread groups of insecticides and have, therefore, been extensively studied in the past decade [[29], [30], [31], [32], [33]]. Like all other insecticides, neonicotinoids and fipronil can have lethal and sublethal impacts on non-target organisms, including insect predators and vertebrates. Furthermore, insecticides often show a wide range of synergistic effects with other stressors [33]. Lately, an increasing amount of research has been led on studying the effects of insecticides on non-target beneficial insects (mainly bees) [29,34,35].

Similarly, research into the efficiency and use of anti-mosquito insecticides or repellents has a long history [36,37]. The reason here is plain enough: mosquitos are vectors for dangerous pathogens and parasites are a key threat for millions of people worldwide. Vector control is, thus, a crucial part of the global strategy for the management of mosquito-associated diseases, and insecticide application is still the most important component in this effort [38,39]. However, with an intensification of the effort for disease control, mosquitos developed resistance to commonly used insecticides [[39], [40], [41]] and the fight against the disease spreading has to be re-evaluated. Benelli [38] has seen the biggest promise in the behavior-based control programs, with special reference to “boosted SIT” (sterile insect technique) and the mosquitocidal botanicals. Benelli and Beier [42] have highlighted the importance of optimization of the methods for vector control, evaluation of the field suitability and efficacy of new mosquito control tools, monitoring of vector populations and environmental changes, which potentially affect the populations, and emphasized on the development of effective and eco-friendly tools to reduce the burden of mosquito-borne diseases.

The scope of *environmental entomotoxicology* also includes studies focusing on the bioaccumulation of heavy metals in insect bodies. Some studies traditionally focus on the evaluation of the accumulation in insects [43], while other works emphasize insects as bioindicators of environmental pollution [1]. Recently, there is an increased effort in research focused on the transfer of different heavy metals via the whole terrestrial [44,45] and aquatic [46,47] food chains (including insects). The effects of certain heavy metal on fitness traits of insect species have been studied sporadically [48].

### 1.4 New trends

In recent years, some new directions for research in *entomotoxicology* have occurred. With an increasing market for sustainable and eco-friendly sources of high-quality animal protein nutrition, insects are of particular interest [49]. Within the European Union, any new food ingredient has to meet certain legal conditions in terms of food safety [50,51]. During the production of insects, there is a possibility of substrate contamination, which can lead to bioaccumulation of xenobiotics in insect bodies. There is, therefore, an increased research focus on such scenarios [50,[52], [53], [54]].

Similarly, there are attempts to rear certain species of insects (e.g. black soldier fly (*Hermetia illucens*)) as animal feed, which could help with the anticipated increase of 60–70% in consumption of animal products by 2050 [53,55,56]. The safety of the rearing has recently been investigated due to the potential of toxic heavy metal accumulation in the insect bodies. Proc et al. [53] found 15 different elements accumulated in *H. illucens* and they recommend this subject to be more focused on. The black soldier fly is becoming popular also for its ability to process organic waste since the larvae accept a great variety of decaying organic matter [57]. Currently, there are almost 1000 species of insects bred by humans with hundreds being commercially available in the EPPO (European and Mediterranean Plant Protection Organization) region and North America [56].

Another interesting opportunity can be seen in blowfly larvae (“maggot”) therapy. The use of maggots has a long history and has recently gained traction in the medical communities of several countries [[58], [59], [60]]. This method is effective for wound debridement and promotes the growth of granulation tissue. Wound exudate, odor, infection, and pain are reduced by the treatment requiring the decreased need for antibiotics and hospital stay duration. Naturally, the efficiency of current medical treatment using maggots is directly linked to the influence of drugs from the medical treatment of a patient. Patients exposed to the larval therapy are often under the influence of different kinds of medicaments (e.g. opioids, etc.), which can accumulate in the maggots and influence their development. Although this medical discipline is ancient and has been studied in detail since the end of the 1940s, there has not been a lot of research into the influence of drugs on larval development.

## 2 Conclusion

This article offers a new way of looking at *entomotoxicology*. Consideration of the recent literature suggests the scope of the term has widened and a new definition has, therefore, been proposed. *Entomotoxicology* does not consider only medico-legal science and it should not be perceived as such. *Forensic entomotoxicology* and *environmental entomotoxicology* should be distinguished while belonging to the same scope of *entomotoxicology*. Accordingly, the use of the term is recommended for both: 1) research studying the effect of xenobiotics on insects and, 2) research using xenobiotics present in insect bodies as evidence of environmental pollution. The standardization of suggested terminology should lead to new opportunities in collaboration across different scientific fields, which could have been overlooked so far.

*Entomotoxicology* has been in the spotlight of scientific interest for almost a hundred years, while *forensic entomotoxicology* is a relatively new branch of *entomotoxicology*. Review of the contemporary literature has shown that the most studied areas of *environmental entomotoxicology* include fighting mosquitos as the most important vector of malaria, protecting crops from pests without excessive ecological burden, and using insects as the bioindicators of habitat pollution by heavy metals. All of these research directions could benefit from a knowledge crossover with *forensic entomotoxicology* as dealing with the effects of specific xenobiotics on insects is one of its main aims.

Even though *entomotoxicology* is facing criticism for some of its inconsistencies and a lot of work needs to be done to standardize its methodology, *entomotoxicology* as a whole shows great potential in both the environmental and forensic sciences. It would be beneficial to incorporate the methods of *entomotoxicology* in other fields to specify how different kinds of xenobiotics influence the development and fitness of studied organisms. It, thus, becomes strategic to investigate the metabolism and behavior of insects when exposed to specific xenobiotics, because the resultant knowledge can be beneficial not only to *forensic entomotoxicology* and *environmental entomotoxicology,* but can illuminate new alternative and potentially unexplored applications in the medical and industrial spheres.

## Funding

This research did not receive any specific grant from funding agencies in the public, commercial, or not-for-profit sectors.

## Ethical approval

This article does not contain any studies with human participants or animals performed by the author.

## Declaration of competing interest

The author declares no conflict of interest.
